# Long-term systemic outcomes of postmenopausal hormone replacement therapy by age at menopause: a real-world cohort study

**DOI:** 10.1093/hropen/hoag072

**Published:** 2026-08-06

**Authors:** Ting-Fang Lu, Chien-Hsing Lu, Yu-Hsiang Shih, Yen-Fu Chen, Chun-Ting Fan, Shao-Jing Wang, Shih-Tien Hsu, Chih-Ku Liu, Lou Sun, Sheau-Feng Hwang, Tsung-Hsien Lee

**Affiliations:** Department of Obstetrics Gynecology & Women’s Health Taichung Veterans General Hospital, Taichung, Taiwan; Institute of Medicine, Chung Shan Medical University, Taichung, Taiwan; Department of Obstetrics Gynecology & Women’s Health Taichung Veterans General Hospital, Taichung, Taiwan; Institute of Biomedical Sciences, PhD Programme in Translational Medicine and the Rong-Hsing Research Centre for Translational Medicine, National Chung-Hsing University, Taichung, Taiwan; Department of Obstetrics Gynecology & Women’s Health Taichung Veterans General Hospital, Taichung, Taiwan; Department of Public Health, Chung Shan Medical University, Taichung, Taiwan; Department of Obstetrics Gynecology & Women’s Health Taichung Veterans General Hospital, Taichung, Taiwan; Department of Obstetrics Gynecology & Women’s Health Taichung Veterans General Hospital, Taichung, Taiwan; Department of Obstetrics Gynecology & Women’s Health Taichung Veterans General Hospital, Taichung, Taiwan; Department of Obstetrics Gynecology & Women’s Health Taichung Veterans General Hospital, Taichung, Taiwan; Centre for General Education, Ling Tung University, Taichung, Taiwan; School of Medicine, China Medical University, Taichung, Taiwan; Department of Obstetrics Gynecology & Women’s Health Taichung Veterans General Hospital, Taichung, Taiwan; Department of Animal Science and Biotechnology, Tung Hai University, Taichung, Taiwan; Department of Obstetrics Gynecology & Women’s Health Taichung Veterans General Hospital, Taichung, Taiwan; Department of Food and Nutrition, Providence University, Taichung, Taiwan; Department of Obstetrics Gynecology & Women’s Health Taichung Veterans General Hospital, Taichung, Taiwan; Department of Palliative Care Unit, Taichung Veterans General Hospital, Taichung, Taiwan; Institute of Medicine, Chung Shan Medical University, Taichung, Taiwan; Department of Obstetrics and Gynecology, Chung Shan Medical University Hospital, Taichung, Taiwan

**Keywords:** postmenopausal, cardiovascular disease, cancer, estrogen, hormone replacement therapy, menopause, progestin, type 2 diabetes mellitus, bone density loss

## Abstract

**STUDY QUESTION:**

Does postmenopausal hormone replacement therapy (HRT), initiated within 1 year after menopause diagnosis, confer differential long-term health effects according to age at menopause?

**SUMMARY ANSWER:**

Long-term postmenopausal HRT was associated with age-dependent clinical outcomes, with broadly consistent cardiovascular and skeletal associations across age groups, but more variable metabolic and oncological associations according to age at menopause.

**WHAT IS KNOWN ALREADY:**

The ‘timing hypothesis’ suggests that HRT initiated closer to menopause may have more favorable cardiometabolic associations than later initiation, but evidence across distinct age-at-menopause strata remains limited.

**STUDY DESIGN, SIZE, DURATION:**

This retrospective new-user cohort study utilized de-identified data from the TriNetX Research Network, which provided access to electronic medical records for ∼111 million patients globally. To establish a relatively healthy baseline cohort, we included women aged 30–90 years without prior major systemic diseases who had a documented diagnosis of menopause. To ensure adequate time for the development of hard clinical endpoints, we required that the index event (initial menopause diagnosis) occurred at least 10 years before data extraction (31 December 2025), thereby guaranteeing a minimum follow-up period of one decade. To reduce immortal time bias and survivor bias inherent in prevalent-user designs, we employed a new-user, time-to-event cohort design. Women with a pre-existing history of type 2 diabetes mellitus (T2DM), cardiovascular disease (CVD), osteoporosis, compression fracture, venous thromboembolism (VTE), breast cancer, or colorectal cancer on or before the index date were excluded.

**PARTICIPANTS/MATERIALS, SETTING, METHODS:**

Women who initiated postmenopausal HRT containing estrogen and/or progestogen within 1 year after menopause diagnosis were propensity score-matched 1:1 with non-users using baseline demographic, clinical, and laboratory covariates. Primary matching yielded 83 670 matched pairs in women who experienced menopause at age ≤60 years and 12 175 matched pairs in women who experienced menopause at age >60 years. Time-to-event analyses using Cox proportional hazards regression were conducted across four clinically defined menopausal age strata: premature ovarian insufficiency (POI, <40 years), early menopause (40–45 years), normal menopause (46–60 years), and late menopause (>60 years). Hazard ratios (HRs) were calculated for incident T2DM, CVD, compression fracture, breast cancer, colorectal cancer, and VTE. Interaction *P*-values were calculated using the normal menopause group (46–60 years) as the reference for interaction testing. Sensitivity analyses were conducted in both the overall ≤60-year and >60-year cohorts using an alternative 1:1 matching strategy based only on age, race, and baseline BMI, excluding laboratory parameters.

**MAIN RESULTS AND THE ROLE OF CHANCE:**

In the overall matched cohort of women experiencing menopause at age ≤60 years, postmenopausal HRT was associated with lower hazards across all evaluated systemic endpoints. Compared with non-users, postmenopausal HRT was associated with lower hazards of T2DM (events: 3517 vs 4580; crude incidence: 4.20% vs 5.47%; HR: 0.595, 95% CI: 0.575–0.615) and CVD (events: 3960 vs 4413; crude incidence: 4.74% vs 5.29%; HR: 0.699, 95% CI: 0.688–0.709). Skeletal health was preserved, evidenced by a lower hazard of compression fractures (events: 426 vs 441; crude incidence: 0.51% vs 0.53%; HR: 0.780, 95% CI: 0.719–0.846). Furthermore, postmenopausal HRT was associated with lower hazards of breast cancer (events: 1281 vs 1504; crude incidence: 1.53% vs 1.80%; HR: 0.786, 95% CI: 0.743–0.831), colorectal cancer (events: 222 vs 276; crude incidence: 0.27% vs 0.33%; HR: 0.847, 95% CI: 0.738–0.971), and VTE (events: 1247 vs 1346; crude incidence: 1.49% vs 1.61%; HR: 0.859, 95% CI: 0.811–0.910). In the primary age-stratified model, the >60-year cohort showed a higher breast cancer hazard (HR: 1.151, 95% CI: 1.057–1.255); in the corresponding >60-year group sensitivity analysis, using alternative matching, the estimate remained directionally similar (HR: 1.218, 95% CI: 1.027–1.444). Across the remaining >60-year sensitivity outcomes, the direction of association was preserved, including continued lower hazards of T2DM, CVD, VTE, and compression fracture, while the colorectal cancer association remained neutral.

**LIMITATIONS, REASONS FOR CAUTION:**

Important limitations include residual confounding, incomplete socioeconomic and screening data, limited details on HRT formulation, route, dose and duration, unavailable breast cancer subtype information, unavailable exact maximum follow-up duration, and the inability to directly incorporate standardized patient-level geography into the matching or regression models. Region-restricted inference was additionally constrained because the Europe, Middle East, and Africa (EMEA) > 60-year HRT cohort contained only 48 exposed users, precluding stable matched analysis.

**WIDER IMPLICATIONS OF THE FINDINGS:**

While cardiovascular and skeletal associations appeared broadly consistent across menopausal ages, the associations with breast cancer in the older age group highlight the critical need for individualized prescribing. These real-world findings are most consistent with earlier initiation of postmenopausal HRT for symptomatic women and those with POI, while urging extreme caution with initiation in late-onset menopause.

**FUNDING:**

No specific external funding was received. The study was supported by departmental funds from the Department of Obstetrics and Gynecology, Taichung Veterans General Hospital.

**DISCLOSURES:**

The authors declare no competing interests.

**TRIAL REGISTRATION NUMBER:**

N/A.

WHAT DOES THIS MEAN FOR PATIENTS? Menopause is a natural transition that can increase long-term risks for health issues such as type 2 diabetes, heart disease, and bone density loss. Although postmenopausal hormone replacement therapy (HRT) is highly effective at relieving menopausal symptoms and protecting bone health, many women and clinicians remain concerned about its long-term safety. To clarify these associations, we studied the health records of a large global network of women over at least a decade to examine how postmenopausal HRT relates to long-term health according to the age at which menopause occurs.

We found that the long-term associations of postmenopausal HRT are not ‘one-size-fits-all’ but depend strongly on age at menopause. Reassuringly, across all age groups, postmenopausal HRT was associated with lower chances of heart disease, bone fractures, and blood clots. However, its association with breast cancer varied substantially by age. For women who experienced menopause prematurely (under age 40), postmenopausal HRT was associated with a lower breast cancer hazard. By contrast, among women who reached menopause after age 60, initiating postmenopausal HRT was associated with a higher breast cancer hazard. Overall, these findings are most consistent with the use of postmenopausal HRT in younger women with early menopause when clinically indicated, while supporting greater caution in women with late-onset menopause.

## Introduction

Menopause represents a physiological transition marked by reduced endogenous estrogen levels, which triggers vasomotor symptoms and increases long-term risk of type 2 diabetes mellitus (T2DM), cardiovascular disease (CVD), and osteoporosis (Pinkerton, 2020; Gartlehner *et al.*, 2022). Hormone replacement therapy (HRT) remains the most effective intervention to alleviate menopausal symptoms and prevent bone loss (Levin *et al.*, 2018; Pinkerton, 2020; Savukoski and Niinimäki, 2025). However, following the publication of the Women’s Health Initiative (WHI) trials (Rossouw *et al.*, 2002), the long-term safety of HRT has been intensely debated, causing widespread prescription hesitancy (Gartlehner *et al.*, 2022; LaMonte *et al.*, 2022; Manson *et al.*, 2024; Schweitzer, 2025).

Subsequent re-evaluations led to the ‘timing hypothesis’, suggesting that early HRT initiation during the menopausal transition confers cardiometabolic protection, whereas late initiation may pose risks (Nudy *et al.*, 2019; El Khoudary *et al.*, 2020; US Preventive Services Task Force, 2022). Nevertheless, the timing hypothesis is not universally accepted, with discrepancies between observational studies and randomized controlled trials (RCTs) (Rossouw *et al.*, 2002; Harman *et al.*, 2005; Hodis *et al.*, 2015, 2016; Miller *et al.*, 2019; Langer *et al.*, 2021). Contemporary trials evaluating early intervention often rely on intermediate surrogate markers due to logistical challenges associated with long-term follow-up for hard clinical endpoints (Harman *et al.*, 2005; Hodis *et al.*, 2015, 2016; Miller *et al.*, 2019). Most trials have focused on women undergoing menopause at an average age (median ∼51 years), leaving clinical uncertainties for those with early (≤ 45 years) and late-onset (>60 years) menopause (Mishra *et al.*, 2024; Savukoski and Niinimäki, 2025).

Epidemiologically, premature ovarian insufficiency (POI, <40 years) and early menopause (40–45 years) affect ∼1–4% and 5–10% of women globally, respectively (Stuenkel and Gompel, 2023; Mishra *et al.*, 2024). Late-onset menopause after age 60 is rare, representing less than 1% of females (Gold, 2011). These extreme age strata hold profound pathophysiological significance. Women with late-onset menopause have experienced prolonged lifetime exposure to endogenous estrogens, delaying the onset of osteoporosis but elevating the risk for hormone-dependent malignancies (Collaborative Group on Hormonal Factors in Breast Cancer, 2012). Consequently, introducing late-life exogenous HRT to aging breast tissue and a potentially atherosclerotic vascular endothelium may paradoxically act as a disease promoter rather than a protector (Chlebowski *et al.*, 2020; Santen *et al.*, 2020).

To address these evidence gaps, we utilized the TriNetX global research network to conduct a large-scale, new-user cohort study, evaluating the associations between long-term postmenopausal HRT use and incident metabolic, cardiovascular, skeletal, and oncological outcomes across menopausal age strata. We hypothesized that the systemic safety and efficacy of postmenopausal HRT are influenced by age at menopause.

## Materials and methods

### Data source and study population

This retrospective, multi-institutional cohort study utilized de-identified data from the TriNetX Research Network, which provided access to electronic medical records for ∼111 million patients globally. To establish a healthy baseline cohort, we included female patients currently aged 30–90 years without a history of major systemic diseases who had a menopause diagnosis. To ensure adequate time for long-term hard clinical endpoints, we strictly required that the index event (initial menopause diagnosis) occurred at least 10 years before data extraction (31 December 2025), guaranteeing a minimum follow-up of one decade. To eliminate immortal time and survivor biases, we employed a new-user, time-to-event cohort design.

The index date was defined as the date of initial menopause diagnosis. Patients were categorized into two cohorts: (1) the HRT group, defined as patients who initiated postmenopausal HRT containing estrogen and/or progestogen within 1 year following the index date; and (2) the non-HRT group, defined as patients with a menopause diagnosis but never prescribed HRT. Detailed diagnostic, procedural, and medication codes are provided in Supplementary Table S1.

### Exclusion criteria

To ensure the assessment of incident outcomes that developed strictly after the onset of menopause, we excluded patients with a history of T2DM, CVD, osteoporosis, compression fracture, VTE, breast cancer, or colorectal cancer documented before or on the index date (Supplementary Table S1).

### Study design and stratification

To investigate age-dependent associations, we stratified the study population into four clinically defined subgroups based on age at the index date: premature ovarian insufficiency (<40 years), early menopause (40–45 years), normal menopause (46–60 years), and late menopause (>60 years).

### Statistical analysis

To minimize indication confounding and healthy user bias, we performed 1:1 propensity score matching (PSM). To prevent over-adjustment for mediators on the causal pathway, PSM was adjusted using baseline (pre-exposure) covariates recorded on or before the index date, including age at menopause, race, baseline BMI, and baseline laboratory parameters, namely creatinine, estimated glomerular filtration rate, alanine aminotransferase, glycated hemoglobin A1c (HbA1c), hemoglobin, triglycerides, low-density lipoprotein cholesterol, high-density lipoprotein cholesterol, and total cholesterol (Supplementary Figs S1 and S2). Missing baseline covariates were handled using complete case analysis during PSM. A standardized mean difference (SMD) < 0.1 indicated negligible baseline imbalance.

Time-to-event analyses used Cox proportional hazards regression models to calculate hazard ratios (HRs) and 95% CIs. The proportional hazards assumption was verified before analysis. Patients were followed from index date until outcome event, death, or last clinical encounter (censoring). Compression fractures were specifically selected over asymptomatic osteoporosis screening as a hard clinical endpoint to minimize surveillance bias.

To evaluate heterogeneity of HRT effects across age groups, interaction *P*-values were calculated using the *Z*-test for differences between regression coefficients, with the normal menopause group (46–60 years) as reference. Given the exploratory, hypothesis-generating nature of analyzing multiple systemic endpoints in a real-world setting, all outcomes were designated as co-primary endpoints. Although multiple testing corrections (e.g. Bonferroni) were not strictly applied, exact *P*-values and 95% CIs are reported for clinical interpretation. A two-sided *P*-value < 0.05 was considered statistically significant.

### Sensitivity analysis

To test the robustness of our primary findings and address concerns regarding the potential over-adjustment of intermediate laboratory variables, sensitivity analyses were conducted in both the overall cohort aged ≤60 years and the cohort aged >60 years. We employed an alternative matching strategy by performing 1:1 PSM strictly based on only demographic factors (age and race) and baseline BMI, intentionally excluding all laboratory parameters. The sensitivity analyses are presented in Supplementary Figs S3 and S4.

## Results

### Study population and baseline characteristics

For women who experienced menopause at age ≤60 years, applying 1:1 PSM based strictly on pre-exposure covariates established a balanced cohort of 83 670 matched pairs. All covariates achieved a standardized mean difference (SMD) < 0.1, indicating negligible baseline imbalance (Table 1). Similarly, for women who experienced menopause at age >60 years, the 1:1 PSM algorithm successfully generated 12 175 matched pairs, demonstrating excellent comparability (Table 2).

**Table 1.** Characteristics of hormone replacement therapy (HRT) users and non-users among women who experienced menopause at age ≤ 60 years.

	Before matching			After matching		
	HRT, N = 84 970	Non-HRT, N = 927 329	SMD	HRT, N = 83 670	Non-HRT, N = 83 670	SMD
Menopause age* (Mean ± SD)	48.1 ± 8.4	51.9 ± 6.2	0.521	50.2 ± 8.9	50.1 ± 9.6	0.009
Race*						
White	78.0	71.3	0.154	77.8	77.6	0.003
Black/African American	10.8	7.3	0.124	7.4	7.3	0.004
Asian	2.3	3.1	0.054	2.3	2.2	0.005
Other race	3.0	2.7	0.007	2.8	2.9	0.006
Unknown race	5.9	15.6	0.238	9.7	10.0	0.028
BMI*	28.5 ± 6.9	27.8 ± 6.3	0.112	27.2 ± 5.4	27.5 ± 5.1	0.047
Creatinine, mg/dl*	0.8 ± 0.7	0.9 ± 0.6	0.077	0.8 ± 0.6	0.8 ± 0.4	0.013
eGFR, ml/min/1.73 m²*	78 ± 24	79 ± 23	0.027	78 ± 24	79 ± 21	0.048
ALT, IU/l*	20.2 ± 15.7	21.4 ± 16.6	0.073	20.2 ± 15.7	20.6 ± 16	0.024
HbA1c, %*	5.5 ± 0.5	5.4 ± 0.6	0.101	5.4 ± 0.7	5.4 ± 0.5	0.046
Hemoglobin, g/dl*	12.5 ± 1.4	12.5 ± 1.6	0.047	12.5 ± 1.4	12.5 ± 1.5	0.044
Triglycerides, mg/dl*	112.7 ± 69.6	108.9 ± 73.9	0.053	112.5 ± 69.5	109.4 ± 78.5	0.042
LDL-C, mg/dl*	115.4 ± 33.6	114.3 ± 32.7	0.031	114.9 ± 33.0	114.5 ± 32.7	0.013
HDL-C, mg/dl*	60.4 ± 18.4	61.8 ± 18.6	0.071	60.5 ± 18.3	61.8 ± 18.6	0.075
Total cholesterol, mg/dl*	197.9 ± 39.1	197.0 ± 41.2	0.021	198.1 ± 39.1	196.5 ± 40.0	0.039

Data are presented as mean ± SD or percentages. Patient characteristics were documented at the index date. Variables marked with * were selected for PSM.

HRT, hormone replacement therapy; PSM, propensity score matching; SMD, standardized mean difference; eGFR, estimated glomerular filtration rate; ALT, alanine transaminase; HbA1c, glycated hemoglobin A1c; LDL-C, low-density lipoprotein cholesterol; HDL-C, high-density lipoprotein cholesterol.

**Table 2.** Characteristics of hormone replacement therapy (HRT) users and non-users among women who experienced menopause at age > 60 years.

	Before matching			After matching		
	HRT, N = 12 375	Non-HRT, N = 1 020 210	SMD	HRT, N = 12 175	Non-HRT, N = 12 175	SMD
Menopause age* (Mean ± SD)	65.3 ± 5.2	66.6 ± 6.9	0.222	65.3 ± 5.2	66.0 ± 6.9	0.071
Race*						
White	78.7	75.0	0.081	78.8	77.9	0.020
Asian	6.7	5.3	0.061	6.7	7.1	0.017
Black/African American	1.7	2.3	0.040	1.7	1.8	0.004
Other race	2.9	3.0	0.007	2.9	2.9	0.000
Unknown race	10.0	14.4	0.138	10.0	10.3	0.017
BMI*	27.0 ± 6.1	29.3 ± 7.2	0.341	27.0 ± 6.1	27.3 ± 6.4	0.047
Creatinine, mg/dl*	1.0 ± 0.8	0.9 ± 0.8	0.015	1.0 ± 0.8	0.9 ± 0.7	0.012
eGFR, ml/min/1.73 m²*	76 ± 24	78 ± 23	0.027	76 ± 24	77 ± 21	0.041
ALT, IU/l*	22.5 ± 22.3	23.1 ± 26.4	0.025	22.5 ± 22.3	22.2 ± 18.2	0.015
HbA1c, %*	5.76 ± 1.0	5.88 ± 1.1	0.115	5.76 ± 1.0	5.77 ± 1.0	0.027
Hemoglobin, g/dl*	12.7 ± 1.6	12.7 ± 1.8	0.048	12.7 ± 1.6	12.7 ± 1.7	0.045
Triglycerides, mg/dl*	112.6 ± 72.6	109.2 ± 69.2	0.049	109.1 ± 69.0	107.5 ± 67.6	0.023
LDL-C, mg/dl*	116.2 ± 33.4	113.1 ± 34.0	0.092	114.5 ± 33.8	113.1 ± 34.0	0.043
HDL-C, mg/dl*	57.8 ± 21.4	62.5 ± 20.9	0.220	61.0 ± 21.7	62.5 ± 20.9	0.069
Total cholesterol, mg/dl*	198.7 ± 39.2	198.1 ± 41.0	0.015	199.0 ± 40.0	198.1 ± 41.0	0.022

Data are presented as mean ± SD or percentages. Patient characteristics were documented at the index date. Variables marked with * were selected for PSM.

HRT, hormone replacement therapy; PSM, propensity score matching; SMD, standardized mean difference; eGFR, estimated glomerular filtration rate; ALT, alanine transaminase; HbA1c, glycated hemoglobin A1c; LDL-C, low-density lipoprotein cholesterol; HDL-C, high-density lipoprotein cholesterol.

### Overall clinical outcomes in women ≤60 years

**Figure 1.**
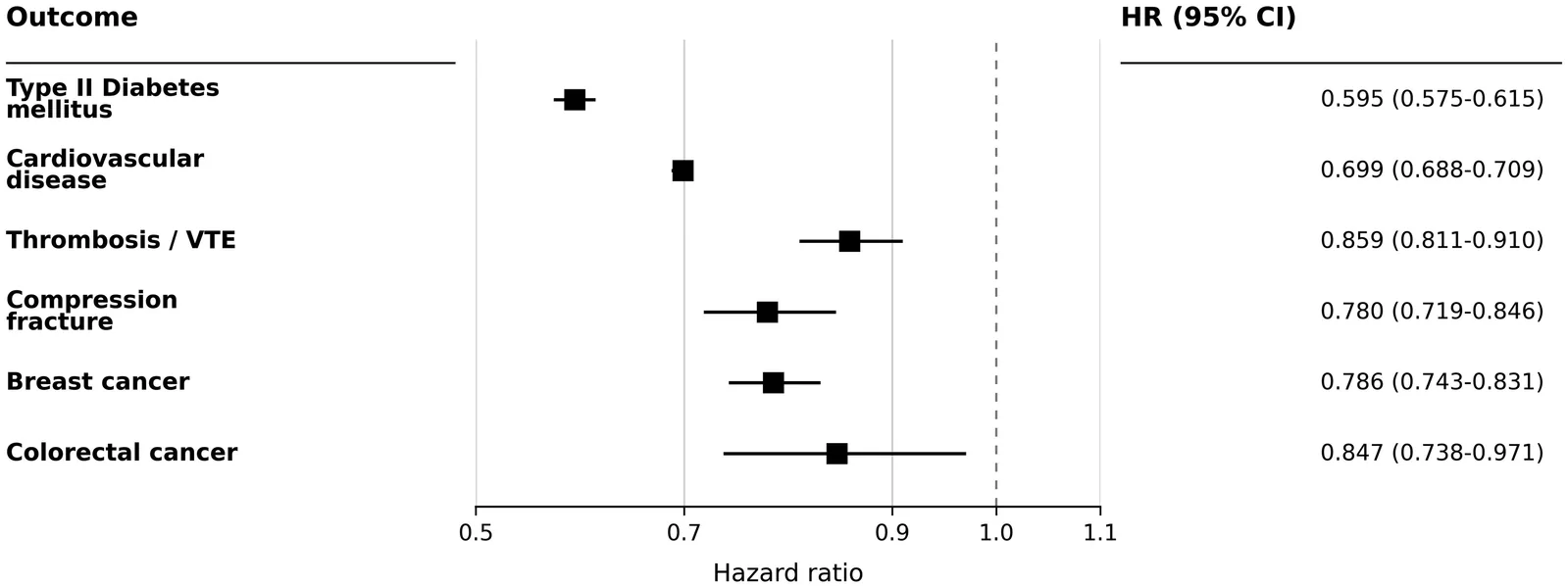
**Overall clinical outcomes associated with postmenopausal hormone replacement therapy in women with menopause at age ≤60 years.** Forest plot displaying the hazard ratios (HRs) and 95% CIs for incident clinical endpoints: type 2 diabetes mellitus (T2DM), cardiovascular disease (CVD), venous thromboembolism (VTE), compression fracture, breast cancer, and colorectal cancer. Postmenopausal hormone replacement therapy (HRT) users were compared with non-users in a 1:1 propensity score-matched cohort of women who experienced menopause at or before age 60 years. Black squares represent point estimates of the HRs and horizontal lines represent the 95% CIs. HR, hazard ratio.

In the matched cohort of women experiencing menopause at age ≤60 years, postmenopausal HRT use was associated with lower hazards across all evaluated systemic endpoints (Fig. 1). Detailed absolute event counts and crude incidence rates for all outcomes are summarized in Supplementary Table S2.

Compared to non-use, postmenopausal HRT use was associated with lower hazards of T2DM (events: 3517 vs 4580; crude incidence: 4.20% vs 5.47%; HR: 0.595, 95% CI: 0.575–0.615) and CVD (events: 3960 vs 4413; crude incidence: 4.74% vs 5.29%; HR: 0.699, 95% CI: 0.688–0.709). Skeletal health was preserved, evidenced by a lower hazard of compression fractures (events: 426 vs 441; crude incidence: 0.51% vs 0.53%; HR: 0.780, 95% CI: 0.719–0.846). Furthermore, postmenopausal HRT was associated with lower hazards of breast cancer (events: 1281 vs 1504; crude incidence: 1.53% vs 1.80%; HR: 0.786, 95% CI: 0.743–0.831), colorectal cancer (events: 222 vs 276; crude incidence: 0.27% vs 0.33%; HR: 0.847, 95% CI: 0.738–0.971), and VTE (events: 1247 vs 1346; crude incidence: 1.49% vs 1.61%; HR: 0.859, 95% CI: 0.811–0.910).

### Age-stratified clinical outcomes and interaction analyses

**Figure 2.**
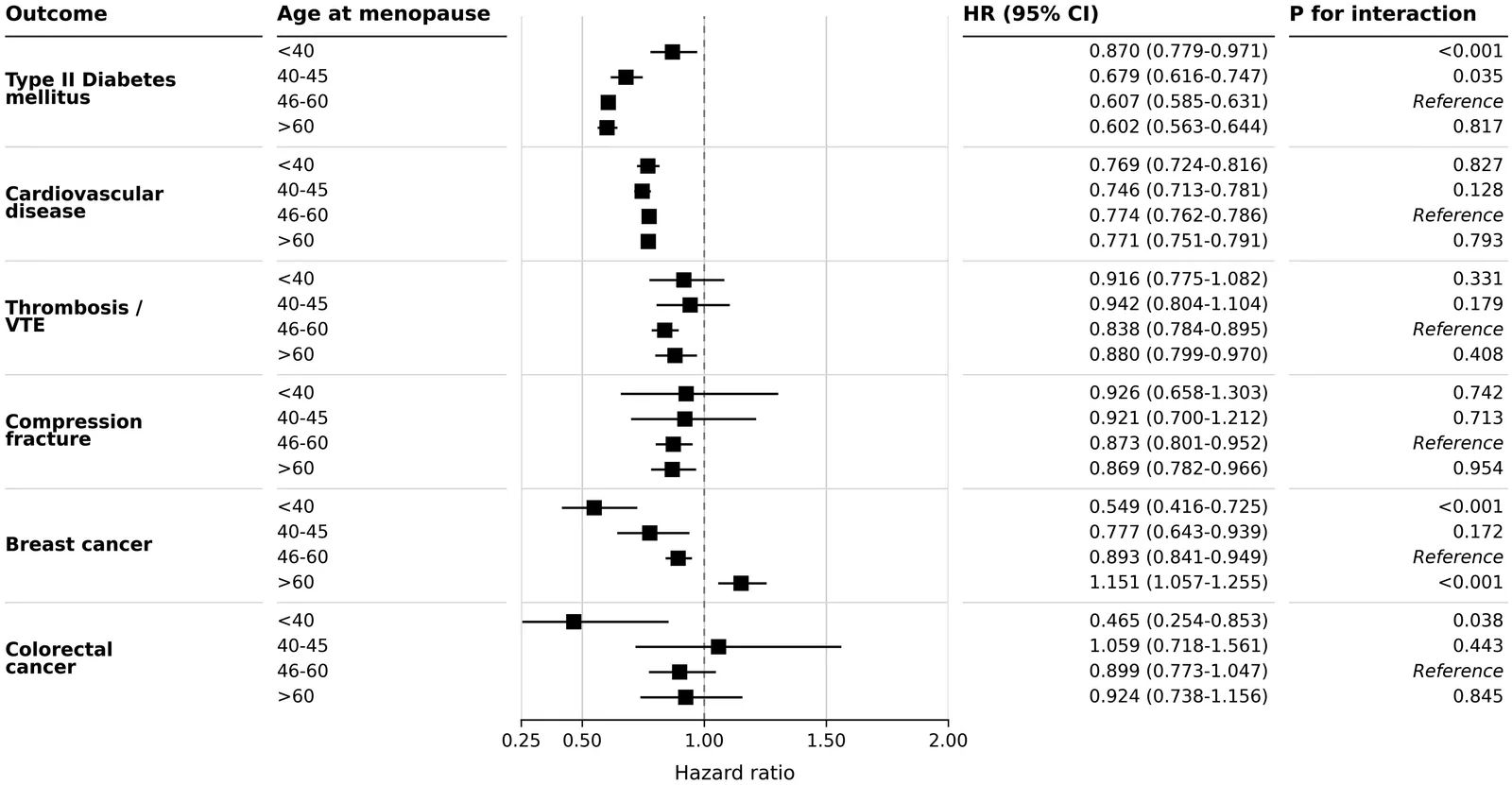
**Age-stratified clinical outcomes and interaction analyses for postmenopausal hormone replacement therapy.** Forest plot illustrating the hazard ratios (HRs) and 95% CIs for incident clinical outcomes associated with postmenopausal hormone replacement therapy (HRT), stratified into four clinically defined age-at-menopause cohorts: <40 years, 40–45 years, 46–60 years, and >60 years. Interaction *P*-values were calculated using *Z*-tests for differences between regression coefficients, with the normal menopause group (46–60 years) as the reference category. Black squares represent the point estimates of the HRs and horizontal lines represent the 95% CIs. VTE, venous thromboembolism; HR, hazard ratio.

Time-to-event analyses were stratified into four menopausal age groups, with the 46- to 60-year group serving as the reference for interaction testing (Fig. 2). Absolute event counts and crude incidences for each specific age stratum are detailed in Supplementary Table S2. Unless otherwise stated, HR estimates in this section refer to the primary age-stratified model shown in Fig. 2; the separate >60-year sensitivity estimate for breast cancer (HR 1.218) is reported in the Sensitivity Analysis section and in Supplementary Fig. S4.

#### Cardiovascular, skeletal, and VTE outcomes

HRT demonstrated consistent protective associations for CVD across all age groups, with HRs ranging from 0.746 (40–45 years) to 0.774 (46–60 years). Interaction analyses showed no significant differences between age strata (all *P for interaction* > 0.05). HRT also provided uniform protection against compression fractures (HRs ranging from 0.869 to 0.926) and VTE risk (HRs ranging from 0.838 to 0.942), without significant age-related interactions (Fig. 2).

#### Metabolic outcomes (T2DM)

The protective association against T2DM was strongest in the reference 46–60 year group (HR: 0.607) and the >60 year group (HR: 0.602). Conversely, this protective effect was significantly attenuated in both the <40 years group (HR: 0.870, *P for interaction* < 0.001) and the 40–45 years group (HR: 0.679; *P for interaction *= 0.035) (Fig. 2).

#### Oncological outcomes

The association between postmenopausal HRT and breast cancer varied by age. In the primary age-stratified model, the 46- to 60-year cohort showed a modestly lower breast cancer hazard (HR: 0.893), whereas the <40-year cohort showed a more pronounced lower hazard (HR: 0.549; *P for interaction* < 0.001), while the >60-year cohort showed a higher breast cancer hazard (HR: 1.151, 95% CI 1.057–1.255; *P for interaction* < 0.001). For colorectal cancer, a lower hazard was observed only in the <40-year group (HR: 0.465; *P for interaction *= 0.038) (Fig. 2).

### Sensitivity analysis

The sensitivity analysis conducted in the overall ≤60-year cohort confirmed the robustness of our primary findings. By matching solely on demographics and BMI without laboratory parameters, postmenopausal HRT remained associated with lower hazards of T2DM (HR: 0.599, 95% CI: 0.579–0.620), CVD (HR: 0.698, 95% CI: 0.688–0.708), VTE (HR: 0.859, 95% CI: 0.811–0.910), compression fractures (HR: 0.780, 95% CI: 0.719–0.846), breast cancer (HR: 0.786, 95% CI: 0.743–0.832), and colorectal cancer (HR: 0.847, 95% CI: 0.738–0.971), with effect sizes nearly identical to the primary model (Supplementary Fig. S3). In the >60-year cohort, the same alternative matching strategy yielded directionally similar findings: T2DM HR: 0.669 (95% CI: 0.594–0.754), CVD HR: 0.838 (95% CI: 0.754–0.929), VTE HR: 0.895 (95% CI: 0.782–0.979), compression fracture HR: 0.845 (95% CI: 0.626–0.903), breast cancer HR: 1.218 (95% CI: 1.027–1.444), and colorectal cancer HR: 0.834 (95% CI: 0.550–1.265) (Supplementary Fig. S4). Thus, the higher breast cancer hazard observed in the primary >60-year model (HR: 1.151) remained evident in the alternative-matching sensitivity analysis.

## Discussion

### Principal findings

In this large real-world cohort study utilizing a new-user design, we demonstrated that the associations between postmenopausal HRT and long-term clinical outcomes are highly dependent on the age at menopause. While postmenopausal HRT was consistently associated with cardiovascular safety, avoidance of VTE and skeletal protection across all age strata, its metabolic and oncological associations varied by age. Notably, breast cancer showed a marked age-dependent contrast, with a lower hazard in early menopause and a higher hazard in women with late menopause.

### Recontextualizing the timing hypothesis and clinical implications

Consistent with previous literature, early and normal-age HRT initiation was associated with cardiovascular and metabolic benefits without increasing VTE risk (Nudy *et al.*, 2019; El Khoudary *et al.*, 2020; US Preventive Services Task Force, 2022). In women experiencing menopause between 46 and 60 years, exogenous estrogen promotes vasodilation and inhibits atherosclerosis (Majmudar *et al.*, 2000; Novella *et al.*, 2019; Somani *et al.*, 2019). Furthermore, HRT prevents the abrupt estrogen decline that triggers insulin resistance (De Paoli *et al.*, 2021; Lambrinoudaki *et al.*, 2022; Cerdas Pérez, 2023; Speksnijder *et al.*, 2023). The absence of elevated VTE risk and the consistent cardiovascular protection observed across all cohorts likely reflect modern prescribing trends, which increasingly favor transdermal estrogens and metabolically neutral progestogens (Stanczyk *et al.*, 2025). Importantly, our study addressed the phenomenon of surveillance bias in skeletal outcomes by utilizing the hard endpoint of compression fractures. This revealed consistent skeletal protection across all age groups (interaction *P*-values > 0.05), emphasizing the importance of selecting objective clinical endpoints to validate the established osteoprotective effects of HRT (Manson and Martin, 2001; Delmas, 2002; Hickey *et al.*, 2005; Gartlehner *et al.*, 2017, 2022; Levin *et al.*, 2018; Eastell *et al.*, 2019; Gosset *et al.*, 2021; Lorentzon *et al.*, 2022; Crandall *et al.*, 2023).

Overall, these patterns in women aged ≤60 years are directionally consistent with the randomized and meta-analytic literature summarized by Hodis and Mack (2022) and provide real-world validation of the broader postmenopausal HRT literature, while still requiring cautious interpretation because this study remains observational.

### Age-dependent metabolic associations

Regarding metabolic outcomes, our study revealed a highly age-dependent protective association against incident T2DM. While robust protection was evident in women undergoing normal or late menopause (HR: ∼0.60), this effect was significantly attenuated in the POI cohort (<40 years, HR: 0.870). This diminished preventive efficacy in younger women may be attributable to several distinct pathophysiological and clinical factors. First, the baseline absolute risk of developing T2DM is inherently low in women below 40. In older cohorts, the rapid estrogen depletion accompanying natural menopause acts synergistically with age-related β-cell senescence to drive visceral adiposity and insulin resistance: a trajectory effectively intercepted by HRT (Mauvais-Jarvis *et al.*, 2013; Roeters van Lennep *et al.*, 2016; Yan *et al.*, 2019). Conversely, in the POI cohort, the minimal baseline event rate mathematically blunts the relative risk reduction (HR) achievable with HRT. Furthermore, the etiology of POI is highly heterogeneous, often involving autoimmune polyendocrine syndromes or genetic aberrations (Maclaran and Panay, 2015; Roeters van Lennep *et al.*, 2016). These underlying conditions may confer independent metabolic and endothelial dysfunctions that standard estrogen replacement cannot fully reverse. Second, a critical discrepancy may exist between standard clinical HRT dosages and the physiological requirements of younger women. Healthy women in their thirties naturally maintain high, cyclically fluctuating serum estradiol concentrations, which are vital to optimize peripheral insulin sensitivity (Mauvais-Jarvis *et al.*, 2013). In real-world practice, patients with POI are frequently prescribed standard menopausal HRT regimens. While these doses are sufficient to alleviate vasomotor symptoms and preserve bone mineral density in older women, they may fall short of replicating the peak physiological estrogen milieu required to maximize glucose homeostasis in a younger population. Clinically, these findings suggest that standard postmenopausal HRT regimens should not automatically be assumed to represent full physiological replacement in women with POI.

### Age-dependent oncological associations

This interpretation should remain cautious because the TriNetX export used here could not distinguish estrogen-only from combined estrogen–progestin regimens, prior HRT users from first-time users, or breast cancer subtype. This is particularly relevant because WHI-related breast cancer findings differ by regimen, as reviewed by Bluming *et al.* (2023).

The most notable interaction observed was the relationship between postmenopausal HRT and breast cancer. In the primary model, the >60-year cohort showed a higher breast cancer hazard (HR: 1.151, 95% CI: 1.057–1.255), and the corresponding >60-year sensitivity analysis using alternative matching yielded a directionally similar estimate (HR: 1.218, 95% CI: 1.027–1.444), supporting the robustness of this late-menopause signal. By contrast, the pronounced protective association in women experiencing POI (<40 years) should not be interpreted as evidence that postmenopausal HRT actively prevents breast cancer in young women. Rather, it suggests that physiological replacement in a profoundly hypoestrogenic state may not reproduce the higher hazard observed when exogenous hormones are introduced after decades of endogenous estrogen exposure (Collaborative Group on Hormonal Factors in Breast Cancer, 2019; Stuenkel and Gompel, 2023). Nonetheless, this finding remains vulnerable to residual confounding, including baseline healthy-user bias, healthcare-seeking behavior, and unmeasured reproductive factors such as parity and family history of breast cancer. The elevated risk observed in the >60-year cohort aligns with prolonged lifetime estrogen exposure before menopause. Introducing exogenous hormones to aging breast tissue that has sustained decades of estrogenic stimulation may promote subclinical micro-carcinomas, manifesting as the higher clinical hazard observed in our study (Collaborative Group on Hormonal Factors in Breast Cancer, 2012; Chlebowski *et al.*, 2020; Santen *et al.*, 2020).

Regarding colorectal cancer, while a protective association was uniquely observed in the <40-year group, neutral effects in older age strata suggest that HRT should not be prescribed solely for primary colorectal cancer prevention (Rennert *et al.*, 2009; Lin *et al.*, 2012; Barzi *et al.*, 2013; Rudolph *et al.*, 2013; Pines, 2016; Topi *et al.*, 2017; Amitay *et al.*, 2020; Nakhostin *et al.*, 2021; Yuk *et al.*, 2024).

### Overcoming the methodological constraints of modern RCTs

Despite the timing hypothesis consensus, modern RCTs encounter logistical and financial challenges in tracking large cohorts of healthy women over the decades required to observe hard clinical endpoints like myocardial infarction or stroke. Consequently, contemporary trials, such as the Kronos Early Estrogen Prevention Study (Harman *et al.*, 2005; Miller *et al.*, 2019) and the Early versus Late Intervention Trial with Estradiol (Hodis *et al.*, 2015, 2016), have relied on intermediate surrogate markers like carotid intima-media thickness with relatively short follow-up durations. Because these short-term trials may not capture the development of hard cardiovascular endpoints, calculating true HRs for these events remains challenging. By utilizing a 111-million-patient global network, our study overcomes these historical constraints, extending beyond surrogate markers to deliver long-term HRs for incident T2DM, breast cancer, and compression fractures over a minimum of a decade of follow-up.

### Strengths and limitations

The strengths of this study include the massive sample size, long-term follow-up, and the use of a new-user, time-to-event design, which eliminated immortal time bias. By strictly matching on pre-exposure covariates and replacing soft diagnostic codes with hard clinical endpoints, we minimized indication confounding and surveillance bias.

However, certain limitations warrant consideration. First, as an observational study, residual confounding from unmeasured variables (e.g. socioeconomic status and healthcare access differences) cannot be entirely ruled out, and healthy user bias may still exert partial influence. Specifically, the TriNetX database lacks comprehensive data on socioeconomic status, lifestyle factors, parity, family history, and individual cancer screening behaviors, all of which could independently influence both HRT prescription and oncological risks. Second, because postmenopausal HRT initiation was defined within 1 year after a menopause diagnosis, the dataset does not allow complete separation of age at menopause from age at HRT initiation. Third, the TriNetX database lacks granular details regarding specific formulations, routes of administration (oral vs transdermal), exact dosages, cumulative duration of therapy, and the crucial distinction between estrogen-only versus combined estrogen–progestin regimens. These variations are critical modulators of both cardiovascular and breast cancer associations. Fourth, standardized patient-level country or geographic-region indicators were not available as covariates in the analytic workflow used for this study; accordingly, geography could not be directly incorporated into the matching or regression models. In addition, the EMEA >60-year HRT cohort was too small (n = 48 exposed users) to support stable matched inference, although an exploratory US-only restriction of the >60-year cohort yielded directionally consistent estimates. Fifth, breast cancer subtype information was not available. Sixth, although all included patients had at least 10 years of follow-up by design, the exact maximum follow-up duration could not be directly exported from the current interface used for this study. Finally, while we have provided absolute event numbers and crude incidences (Supplementary Table S2) to aid clinical interpretation, calculating precise absolute risk differences and incidence rates per 1000 person-years based strictly on propensity score-matched cohorts was precluded due to data extraction constraints. Because HRT users typically maintained longer follow-up durations (person-years) than non-users, comparing crude event counts directly introduced significant time-at-risk bias. Therefore, we utilized robust, time-adjusted Cox model HRs as our primary measure.

## Conclusion

By securing a minimum follow-up of one decade, this study addresses the intrinsic limitations of short-term randomized trials in evaluating true hard clinical endpoints. Long-term postmenopausal HRT is associated with substantial age-dependent variations in health outcomes. While cardiovascular and skeletal safety appear robust across menopausal ages, the breast cancer and T2DM findings underscore the need for individualized prescribing. Overall, these real-world findings are most consistent with earlier initiation of postmenopausal HRT for symptomatic women and those with POI, while supporting greater caution in late-onset menopause cases.

## Supplementary Material

hoag072_Supplementary_Data

## Data Availability

The data underlying the research results described in this article were obtained from the TriNetX Research Network. Access to these de-identified electronic medical records is subject to the data sharing policies and licensing agreements of TriNetX.
