# Supplementary material for: Long-term systemic outcomes of postmenopausal hormone replacement therapy by age at menopause: a real-world cohort study
Source: Hum Reprod Open. 2026 Aug 6;2026(3):hoag072. doi: 10.1093/hropen/hoag072 (PMC13493047; doi:10.1093/hropen/hoag072)
Supplement: Supplementary file 1 [file hoag072_supplementary_data.docx]

**Supplementary Figure S1. Propensity score distributions before and after matching.** (**A**) Distribution before matching and (**B**) distribution after matching for HRT and non-HRT groups aged ≤60 years. In panel B, the purple HRT curve remains visible after matching and closely parallels the green non-HRT curve but is not completely superimposed. HRT, hormone replacement therapy.

**Supplementary Figure S2. Propensity score distributions before and after matching.** (**A**) Distribution before matching and (**B**) distribution after matching for HRT and non-HRT groups aged >60 years. HRT, hormone replacement therapy.

**Supplementary Figure S3. Sensitivity analysis: clinical outcomes in women aged ≤60 years matched strictly without laboratory parameters.** Forest plot showing results of the sensitivity analysis. Patients were 1:1 propensity score matched based solely on demographic factors and baseline BMI, completely excluding intermediate laboratory parameters to address potential over-adjustment bias. The HRs and 95% CIs remained highly consistent with the primary analysis. BMI, body mass index; HR, hazard ratio; CI, confidence interval; VTE, venous thromboembolism.

**Supplementary Figure S4. Sensitivity analysis: clinical outcomes in women with menopause at age >60 years matched without laboratory parameters.** Forest plot showing the >60-year sensitivity analysis. Patients were 1:1 propensity score matched using age, race, and baseline BMI only, excluding laboratory parameters. The pattern of associations remained directionally consistent with the primary analysis, including persistence of the higher breast cancer hazard and a neutral colorectal cancer association. BMI, body mass index; HR, hazard ratio; CI, confidence interval; VTE, venous thromboembolism.
